# Happiness and its Relationship with Job Burnout in Nurses of Educational Hospitals in Tabriz, Iran

**DOI:** 10.30476/IJCBNM.2020.83298.1138

**Published:** 2020-10

**Authors:** Tahere Javadi Sharif, Mina Hosseinzadeh, Nader Mahdavi, Hossein Namdar Areshtanab, Geoffrey L. Dickens

**Affiliations:** 1 Department of Community Health Nursing, School of Nursing and Midwifery, Tabriz University of Medical Sciences, Tabriz, Iran; 2 Department of Epidemiology, Student Research Committee, School of public Health, Iran University of Medical Sciences, Tehran, Iran; 3 Department of Mental Health Nursing, School of Nursing and Midwifery, Tabriz University of Medical Sciences, Tabriz, Iran; 4 Center for Applied Nursing Research (CANR), School of Nursing and Midwifery, Western Sydney University, Sydney, Australia

**Keywords:** Happiness, Iran, Job burnout, Nurses

## Abstract

**Background:**

Happiness is a positive feeling that is vital and significant to maintain health. Nurses are working in difficult conditions which may heavily affect their level of happiness and ability to provide care. Job burnout is a mental reaction against some persistent source of workplace stress. The purpose of this study was to identify happiness and its relationship with job burnout in nurses working at Tabriz’s educational hospitals.

**Methods:**

This descriptive-correlational study was conducted on 344 nurses working at Tabriz’s hospitals in 2018. The subjects were selected by means of proportionate stratified random sampling. Data were collected using three questionnaires (demographic information, job burnout with 22 items and three subscales and Oxford happiness with 29 items) and analyzed in SPSS version16 using descriptive statistics. Statistical tests such as Pearson correlation coefficient, independent t-test, one-way ANOVA, and multiple linear regression analysis were used to analyze the data.

**Results:**

The age range of the participants was 23–57 years with a mean of 35.9±7.5. The mean score of happiness was 64.2±11.5, (score range 35 to 116), which suggests an average level of happiness among the nurses. There was a negative correlation between happiness and total job burnout (r=-0.29, P<0.001). This negative correlation remained significant (B=-0.15, P<0.001) even when nurses’ perception of own health status (B=-5.24, P=0.01), history of illness (B=-4.47, P=0.04), job position (B=-6.61, P=0.001), and type of employment (B=3.56, P=0.03) as potential confounding factors were adjusted.

**Conclusion:**

Considering the reverse relationship between job burnout and happiness, it is suggested that managers try to improve the workplace by managing condition which could lead to job burnout, and therefore use the results to increase the happiness of nurses.

## INTRODUCTION

Happiness can be defined as a positive thinking, psychological comfort, and inner peace, which is vital and significant in maintaining health. ^1^ It can significantly influence the behaviors and performance of employers. Happiness level is important in all professions, especially in nursing where the therapeutic use of self is a key component of the practice. ^2^ Nurses have considerable direct contact with patients who need self-confident, creative, kind and energetic individuals; all of these characteristics are directly related to the feeling of wellbeing. ^1 , 3^ Globally, nurses are working in difficult conditions which may affect their emotional welfare and ability to provide care. ^1^ As one aspect of the nurses’ emotional welfare, happiness has been defined as subjective wellbeing and comprises three major parts: a degree of positive feelings or joy, absence of negative feelings, and an average level of satisfaction at a particular time. ^4^ It also has been described as a parts of a person’s health. ^5 , 6^

Recent studies refer to the importance of nurses’ happiness, arguing that its presence might positively affect their ability to help patients. ^7 , 8^ The consequences of happiness could be higher job performance and satisfaction, better inter-professional relationships, higher patient’s satisfaction and lower hospital costs. ^1^ Recent studies showed that the level of happiness in nurses is low to moderate. ^9 , 10^ Nursing is considered a stressful profession because of the significantly difficult work environment and the nature of the work which requires intricate technical skills, vigilance and judgment. ^11^ The National Institute for Occupational Safety and Health stated that it is anticipated that nurses’ work-related stress will increase in the coming years and this should be addressed because of the deep impact it has in the workplaces as well as later consequences such as job burnout. ^11 , 12^ Job burnout is a mental reaction against some persistent source of workplace stress ^11^ and is more common in healthcare professionals than in other job categories. ^12^ It results from an interaction of multiple factors including personal, interpersonal, and occupational factors; it can have negative effects on nurses, patients and healthcare provider systems. ^13^ For instance, it can affect the quality of nursing care and can lead to job abandonment or work absenteeism. ^14^ A recent study showed that absenteeism results in significant issues such as decreased quality of care and direct costs. ^12^ The cost of the sickness absence for nurses and nursing aides employed at a Portuguese hospital between 2009 and 2013 was 189,679.87 EUR, which was estimated at approximately 10% of total hospital costs. ^15^ A recent UK-study reported that approximately 42% of nurse respondents suffered job burnout. ^16^ Findings of a systematic review suggest that the prevalence of job burnout is around 36% in Iranian nurses. ^14^ Prevention and reduction of job burnout could have positive impacts including the enhanced feeling of happiness. ^11^

Given the inherently stressful nature of nursing, the importance of nurses’ health and the key role of human relationships between nurses and patients, ^15^ and the educational and caring responsibilities of nurses, it is important to pay attention to the nurses’ happiness. There are few studies on nurses’ happiness and its determinant factors, especially in Iran, and the available literature has examined the relationship of some demographic and occupational characteristics with happiness. ^17 , 18^ Some studies have mentioned job burnout as a factor related to happiness, ^11^ but there is little empirical evidence on this issue. The purpose of the current study was to identify happiness and associated factors and its relationship with job burnout in nurses of all educational hospitals in Tabriz.

## MATERIALS AND METHODS

The study used a cross-sectional and correlational survey design. Data were collected from July to December 2018. The study was conducted across all ten educational hospitals of Tabriz. The sampling frame consisted of the nursing staff (N=2600) employed at any of the educational hospitals in 2018. Required sample size was calculated to be 312, (Confidence Interval (CI) of 95%, test power of 80%) based on Hosseini et al. study on the relationship between job burnout and performance of clinical nurses in Shiraz, ^19^ but given the availability of subjects and the possible non-response rate of 15%, the required sample size was ultimately raised to 358.

$n={\left[\frac{\left(Z_{\mathrm{\alpha /2}}+Z_{\beta }\right)}{\mathrm{0.5}\times \mathrm{1n}[\mathrm{(1+r)}/\mathrm{(1-r)}]}\right]}^{2}+3$

α=0.05, β=0.20, r=0.16

$\mathrm{n0}={\left[\frac{\left(\mathrm{1.96}+\mathrm{1.28}\right)}{\mathrm{0.5}\times \mathrm{1n}[\mathrm{1.16}/\mathrm{0.84}]}\right]}^{2}+3=304$

The final volume: R^2^=(0.16)^2^

$N_{i}=\frac{\mathrm{n0}}{1-R^{2}}=\frac{\mathrm{304}}{1-{\mathrm{0.16}}^{2}}=312$

Sampling method was proportionate stratified random sampling, conducted randomly using a random number table and stratified by hospital and ward. Inclusion criteria were being nurse in direct patient care units and willingness to participate in the study. The nurses who had depression or other psychiatric problems according to self-declaration or responded the questions incompletely were excluded from the study and other individuals were replaced randomly.

Data were collected using three questionnaires:

**Demographic and occupational information**. This tool was purpose-designed for the study and collected information on age, sex, education, shift work (rotating shift), type of employment, work experience, hospital ward, height, weight, physical exercise, and sleeping state.

**Maslach Burnout Inventory (MBI)**. The MBI was developed by Christina Maslach (1981); it is a 22-item measure comprising three subscales: emotional exhaustion (9 items, score ranges 0-54, ≥27 high, 17-26 average, ≤16 low), depersonalization (5 items, score ranges 0-30, ≥13 high, 7-12 average, ≤6 low), and diminished sense of personal accomplishment (8 items, score ranges 0-48, ≥39 high, 32-38 average, ≤31 low). ^20^

Cronbach’s alpha value for the main scale was 0.93, and for the emotional exhaustion subscale, it was 0.91. For the depersonalization subscale it was 0.81, and for the diminished personal accomplishment subscale, it was 0.84. ^13^ Construct validity of the three-dimensional structure of the MBI was convincingly demonstrated by confirmatory factor analysis. ^21^ Results of a recent study showed that Persian version of MBI has sufficient validity and reliability; Cronbach’s alpha for three dimensions was more than 0.7. As to construct validity, the results of explanatory factor analysis showed that the correlation coefficient of each item with its own dimension was higher than 0.4 and above other dimensions. ^22^ Because of the difference in study population, face and content validity of the questionnaire was determined before the study; 10 faculty members of Tabriz University of Medical Sciences evaluated the questionnaire and their comments were applied in the questionnaire. The whole instrument had an appropriate Cronbach alpha equal to 0.85.

**Oxford Happiness Inventory (OHI)**. It was developed by Hills and Argyle (2002). It is a 29 item tool. The items were scored from 0-3, constructed to reflect incremental steps defined as unhappy or mildly depressed, a low level of happiness, a high level of happiness, and mania, and the minimum and maximum scores were 0-87 respectively, with the score of 0 indicating the lowest level of happiness and 87 representing the highest level of happiness. There is no cut off point for this tool. Validity and reliability of the main scale was determined in a recent study. Using explanatory and confirmatory factor analysis the construct validity of OHI was confirmed. Cronbach’s alpha value for the main scale was 0.90, ^23^ and for the Persian version of the tool it was 0.93. Findings of a recent study confirmed the internal reliability, construct, and concurrent validity of the Oxford Happiness Inventory in Iran. ^24^ In order to determine the face and content validity of the questionnaire in the present study, 10 faculty members of Tabriz University of Medical Sciences evaluated the questionnaire and their comments were applied in the questionnaire. To determine the reliability of the questionnaire, Cronbach’s alpha coefficient for the instrument was calculated. The whole instrument had an appropriate Cronbach alpha equal to 0.89.

Normal distribution of the quantitative data was verified by the Kolmogorov-Smirnov test. The data were analyzed in SPSS version 16 using descriptive statistics (mean, standard deviation and relative frequency) and statistical tests such as Pearson correlation coefficient, independent sample t-test, one-way ANOVA, and multiple linear regression analysis.

The research proposal was approved by the Ethics Committee of IRB Medical Sciences (ethical code: IR.TBZMED.REC.1397.330). The research goals, anonymity of the information provided and voluntary participation were first explained and the participants then read and signed the written informed consent form; then, they completed and returned the questionnaires.

## RESULTS

In total, 344 nurses participated in the research (Response rate=96%). Participants were aged 23-57 years (mean age: 35.9±7.5). 13.4% of them were male and 86.6% were female. Demographic and organizational information of the participants is summarized in Table 1. Mean scores from the MBI and OHI are shown in Table 2.

**Table 1:** Frequency distribution of the demographic and organizational information (total=344)

Personal information	Number (%)	Personal information	Number (%)	Personal information	Number (%)
Sex		Having second job		History of illness	
Male	46 (13.4)	No	319 (92.7)	No	172 (50.0)
Female	298 (86.6)	Yes	25 (7.3)	Yes	172 (50.0)
Marital status		Job Position		Work shift	
Single	90 (26.2)	Nurse	309 (89.8)	Morning-fixed	75 (21.8)
Married	252 (73.2)	Head nurse	21 (6.1)	Afternoon/night-fixed	14 (4.1)
Divorced and widowed	2 (0.6)	Supervisor	14 (4.1)	Rotating	255 (74.1)
Education		Type of employment		Body Mass Index (BMI)	
Associate degree	4 (1.1)	Formal	213 (61.8)	Underweight (<18.5)	5 (1.5)
BSc	331 (96.2)	Temporary	69 (20.1)	Normal (18.5-24.9)	178 (51.7)
MSc	9 (2.7)	Contractual	62 (18.1)	Overweight (25.9-29)	125 (36.3)
Income	Obese (30 and above)	36 (10.5)		
Insufficient	122 (35.5)	Hospital ward		Sleeping rate	
Rather sufficient	176 (51.1)	Intensive care	112 (32.6)	Sufficient	88 (25.6)
Sufficient or high	46 (13.4)	ER or clinic	32 (9.3)	Insufficient	229 (66.6)
Whom do you live with		Psychiatry	16 (4.7)		
With spouse	238 (69.2)	Pediatrics	13 (3.8)	No children	144 (41.9)
With parents	71 (20.6)	Internal	60 (17.4)	One	100 (29.1)
Alone	32 (9.3)	Surgery	67 (19.5)	Two	89 (25.9)
With relatives	3 (0.9)	Operating room	27 (7.8)	Three or more	11 (3.2)
Physical exercise		Hematology	17 (4.9)	Age (mean±SD)	35.9±7.1
Less than 30 minutes every other day	208 (60.5)	One’s perception of own health status (compared to peers)		Years of employment (Mean±SD)	11.8±7.1
30 minutes every other day	109 (31.7)	Better	41 (9.11%)		
		Similar	170 (49.4)		
Regular daily exercise	27 (7.8)	worse	132 (38.7)		

**Table 2:** Mean scores and standard deviations of the happiness and dimensions of job burnout

Job burnout and its dimensions	Observed range	Mean±SD
Burnout total score	3-108	66.0±14.5
Emotional exhaustion	0-45	24.4±10.1
Depersonalization	0-25	8.7±4.1
Personal accomplishment	1-42	27.8±5.8
Happiness score	35-116	64.2±11.5

Pearson correlation test was used for identifying the relationship between happiness and the dimensions of job burnout (Table 3) and the results showed significant relationships between happiness and all of the dimensions (P<0.001).

**Table 3:** The relationship between happiness and job burnout and its dimensions

	Total score of happiness in Nurses	
Score of job burnout		
Emotional exhaustion		r=-0.48
		P<0.001
Depersonalization		r=-0.13
		P=0.0=1
Low personal accomplishment		r=0.2
		P<0.001
Total score of job burnout		r=-0.29
		P<0.001

Pearson correlation coefficient, independent t-test, and one-way ANOVA were used to determine significant differences or relationships between OHI scores and the measured demographic and organizational characteristics and burnout scores (Tables 4 and 5). All the variables with P<0.2 in univariate analysis were entered into the multiple linear regression model (income, number of children, job position, type of employment, having second job, nurses’ perception of own health status, history of illness, physical exercise, age, years of employment) and their relationship with happiness, while controlling for the effect of other variables, was determined.

**Table 4:** The relationship between happiness and demographic and organizational characteristics

Demographic and organizational variables		Happiness mean±SDP value	P value	Demographic and organizational variables		Happiness mean±SD	P value
Type of employment	Formal	62.9±11.3	P=0.004*	Sex	Male	64.4±13.6	P=0.87*8
	Temporary	64.4±11.8			Female	64.1±11.2	
	Contractual	69.0±9.8					
Second job	Yes	63.9±11.2	P=0.12**	Marital status	Married	64.0±11.5	P=0.67*
	No	67.6±14.4			Single/divorced/widowed	64.6±11.4	
Hospital ward	Intensive care	63.3±10.9	P=0.71**	Education	Associate degree	73.0±15.6	P=0.55*
	Clinic	62.7±11.2			BSc	64.1±11.5	
					MSc	64.4±11.7	
	Psychiatry or hematology	64.9±14.7					
	Pediatrics/ surgery/internal	65.0±10.7					
Operating room	64.3±14.3					
Nurses’ perception of own health status (compared to peers)	Better	69.9±14.9	P<0.001*	Income	Insufficient	62.8±12.1	P=0.17*
	Similar	65.5±10.7			Rather insufficient	64.5±10.5	
	Worse	60.7±10.3			Sufficient or high	66.4±13.2	
History of illness	No	67.3±11.5	P<0.001*	Whom do you live with?	Spouse	63.9±11.2	P=0.73**
	Joint pain	65.3±10.2			Parents	64.7±11.3	
	One chronic diseases	64.2±8.5			Alone or with relatives	65.3±13.6	
	More than one disease	57.8±10.7			No children	65.4±11.3	
Physical exercise	Less than 30 minutes every other day	63.3±11.7	P=0.21*	Number of children	One	63.0±11.4	P=0.2**
	30 minutes every other day	65.3±10.0			Two or more	63.5±11.7	
	Daily exercise	66.2±14.9					
Mass body Index (BMI)	Underweight (<18.5) or normal (18.25-24.9)	63.8±11.8	P=0.65*	Work shift	Morning-fixed	64.8±11.9	P=0.59**
	25-29.9	64.3±10.4			Afternoon/nigh-fixed or rotating	64.0±11.4	
	Obese (30 or above	65.7±13.6			Nurse	63.9±11.1	P=0.005**
					Head nurse or supervisor	69.4±13.8	
Sleeping state	Sufficient	65.1±11.3	P=0.33**	Job Position	Age	35.9±7.5	P=0.07
	Insufficient	63.7±11.5			Years of employment	11.8±7.1	P=0.06

* One way ANOVA,

** t-test

**Table 5:** Results of the final multiple linear regression analysis model for the factors associated with nurses’ happiness

Potential predictor variables	β Coefficient	Standardized βCoefficient	Standard Error(SE)	T	P value
Constant	83.1	-	3.22	25.74	P<0.001
Job Position					
Head-nurses/ Supervisors	Ref	-	-	-	-
Nurses	-6.61	-0.17	1.92	-3.44	P=0.001
Type of employment					
Formal	Ref	-	-	-	-
Temporary	0.76	0.03	1.49	0.51	P=0.61
Contractual	3.56	0.12	1.61	2.21	P=0.03
Illness history					
No	Ref	-	-	-	-
Joint pain	0.77	0.02	1.97	0.39	P=0.70
Distress	-4.47	-0.11	2.17	-2.06	P=0.04
Other chronic disease	-1.33	-0.03	2.11	-0.63	P=0.53
Disease>1	-5.68	-3.40	1.67	-3.40	P=0.001
Nurses’ perception of own health status (compared to peers)					
Better than them	Ref	-	-	-	-
Like them	-2.60	-0.11	1.84	-1.41	P=0.16
Worse than them	-5.24	-0.22	2.02	-2.60	P=0.01
Job burnout	-0.15	-0.18	0.04	-3.57	P<0.001

The results of multiple linear regression analysis showed that, considering the confounding variables (job position, one’s perception of own health status, type of employment, history of illness), the relationship between job burnout score and happiness score was significant (β=-0.15, P<0.001), (Table 5). Based on this result, with one-point increase in the nurses’ job burnout score, their happiness score decreased by 0.15 on average. Nurses with temporary employment or contracted situation (vs formal situation) had a higher average happiness score (β=3.56; P=0.03). About job position, nurses had lower average happiness score than supervisors or head nurses (β=-6.61; P=0.001). Those who reported having a history of distress or having more than one disease (vs. those who reported no history of illness) had a lower average happiness score (β=-4.47; P=0.04, and β=-5.68; P=0.001), respectively. Also, nurses who reported perception of their own health status worse than their peers had lower happiness score of 5.24 points (β=-5.24, P=0.01). These variables were determinants of happiness score and explained 18.87% (Adjusted R-squared=18.87%) of the variance (variability) of happiness.

## DISCUSSION

The purpose of this study was to explore happiness and identify its relationship with job burnout among the nurses of Tabriz’s educational hospitals. Results showed that most of the subjects had a moderate and low happiness score (less than 65). Findings of a similar study showed a moderate level of happiness among hospital nurses. ^17^ In another study in South Korea, happiness level of nursing students was reported moderate (on a 1-6 scale). ^10^ Most studies have shown that nurses working at hospitals enjoy a low to average level of happiness which could possibly be associated with the negative feelings the nurses experience while taking care of the disabled and patients or with difficult working condition and heavy work load.10, 17

Regarding job burnout variable, it was indicated that there was a rather high level of job burnout among the nurses under the study, and dimension of low personal accomplishment had the higher score among all dimensions, which is in the same line with recent studies in Iran. ^25 , 26^

A significant relationship was found between happiness and the mean score of total job burnout and all of its dimensions. Consistent with the current study, a negative significant relationship was found between total job burnout and positive feeling and subjective wellbeing. ^12 , 27^

No significant relationship was found in this study between happiness and sex or marital status which is consistent with some previous studies. ^18^ One recent study reported that happiness score was significantly higher in married students. ^17^ The difference could be attributed to different research subjects as nurses have less opportunity than students to be with and enjoy their families because of difficult working condition. No significant relationship was found either between the type of the ward and the mean score of happiness, which is also in line with the studies carried out in Iran and Korea. ^28 , 29^

The variables that had a significant relationship in the final model with happiness included job position, type of employment, nurses’ perception of own health status, history of illness and job burnout. In the present study, those who had no history of illness and considered themselves healthier had higher happiness scores. Nurses’ perception of own health status and history of illness have also been reported as significant relevant factors of happiness in a recent study in South Korea, ^29^ which confirms the fact that health related variables affect nurses’ happiness significantly. 

Job position was another factor that had a significant relationship with the nurses’ happiness. Head nurse or supervisors obtained higher happiness score; this finding is consistent with some recent studies. ^4 , 5^ Nurses working at higher levels are considered the pillars of their organization and have more self-control and show more social adaptability. Leadership and integrated management at workplace also play a pivotal role in creating a supportive environment and promoting the feeling of wellbeing and happiness. ^4^

A similar result was found as to the type of employment and its significant relationship with job happiness, ^10^ but no non-Iranian study was found on the issue. The reason might be that the type of employment is usually not varied in other countries. 

Although sufficient for the analytical purposes of this study, obtaining the sample from one city was one of the limitations, but using proportional stratified random sampling in all educational hospitals can be the strengths of the study which can increase the generalizability of the results. Exclusion of other factors including job satisfaction, organizational commitment and personal characteristics which are said to be influential on happiness was another limitation; these variables along with job burnout can predict happiness well. It is, therefore, suggested that future research should take other influential factors into account. A third limitation was the application of self-report instruments that may not be responded correctly by the participants because of inadequate introspection or irresponsible answers to the items.

## CONCLUSION

The results show that the nurses working at Tabriz’s educational hospitals are somewhat unhappy, under the influence of various factors including job position, type of employment, one’s perception of own health status, history of illness, and job burnout. Health managers and policymakers can benefit from these findings to promote the strategies for enhancing the nurses’ happiness and ultimately improving the quality of nursing care. Considering the reverse relationship between job burnout and happiness, we also suggest that managers try to improve the workplace by managing conditions which could lead to job burnout, and, therefore, use the results to increase the happiness of nurses.
